# Perioperative Anemia, Transfusion Practices, and Patient Blood Management: Lessons from the COVID-19 Pandemic

**DOI:** 10.3390/hematolrep18030037

**Published:** 2026-05-30

**Authors:** Alin Ionescu, Alexandra Mihăilescu, Raluca Dumache, Alexandru Capcelea, Alexander Dean Turceanu, Nicolae Albulescu, Mihai Alexandru Săndesc

**Affiliations:** 1 Research Center in Anesthesia and Intensive Care (CCATITM), “Victor Babeș” University of Medicine and Pharmacy, 300041 Timișoara, Romania; ionescu.alin@umft.ro; 2 Centre of Genomic Medicine, Genetics Discipline, “Victor Babeș” University of Medicine and Pharmacy, 300041 Timișoara, Romania; 3 Center for Ethics in Human Genetic Identifications, “Victor Babeș” University of Medicine and Pharmacy, 300041 Timișoara, Romania; raluca.dumache@umft.ro; 4 Doctoral School, “Victor Babeș” University of Medicine and Pharmacy, 300041 Timișoara, Romania; alexandru.capcelea@umft.ro; 5 Medical School, “Victor Babeș” University of Medicine and Pharmacy, 300041 Timișoara, Romania; alexander.turceanu@student.umft.ro; 6 Department of Internal Medicine II, Division of Cardiology, Centre for Molecular Research in Nephrology and Vascular Disease, “Victor Babeș” University of Medicine and Pharmacy, 300041 Timișoara, Romania; nicolae.albulescu@umft.ro; 7 Teodor Șora Research Center, “Victor Babeș” University of Medicine and Pharmacy, 300041 Timișoara, Romania; sandesc.mihai@umft.ro

**Keywords:** perioperative anemia, blood transfusion, patient blood management, iron deficiency, transfusion alternatives, COVID-19

## Abstract

The COVID-19 pandemic exposed vulnerabilities in global blood supply systems and accelerated the adoption of patient blood management (PBM) strategies aimed at optimizing transfusion practices in surgical care. Perioperative anemia is a key contributor to adverse outcomes and is frequently treated with allogeneic blood transfusion (ABT), which carries infectious and immunologic risks. Iron deficiency remains the most common and potentially correctable cause of perioperative anemia. This narrative review examines various approaches to perioperative anemia, strategies to minimize reliance on ABT, and alternatives within the PBM paradigm. Evidence supports the use of iron therapy, erythropoiesis-stimulating agents, antifibrinolytic strategies, and blood conservation techniques to reduce transfusion requirements and improve clinical outcomes. Lessons from the COVID-19 pandemic highlight PBM as a framework to enhance transfusion safety and sustainability. Broader implementation of PBM may improve patient outcomes, reduce unnecessary transfusions, and preserve scarce blood resources.

## 1. Introduction

Global health crises expose structural vulnerabilities in healthcare systems, demanding rapid reallocation of medical resources. One of the sectors most affected during large-scale emergencies is the blood supply system since donor availability declines and access to blood products becomes significantly reduced. The COVID-19 pandemic represented an unprecedented stress test for blood services worldwide and accelerated the implementation of patient blood management (PBM) strategies as a consequence of the marked decline in blood donations [1,2]. These challenges emerged in a context in which anemia already represented a major global health burden. It is estimated that approximately one quarter of the world’s population is affected by anemia, a condition associated with increased morbidity, mortality, and substantial healthcare costs [3]. The pandemic caused widespread societal and healthcare disruptions, placing additional strain on human, material, and economic resources within healthcare systems [4]. The coexistence of a high global prevalence of anemia and restricted blood availability created overlapping pressures on surgical care and transfusion services. Patient blood management (PBM) is increasingly recognized as a comprehensive, evidence-based strategy designed to optimize the care of patients with anemia while minimizing exposure to allogeneic blood transfusion (ABT). Recognized and promoted by the World Health Organization as a framework for improving patient safety and conserving blood resources, PBM is structured around three interrelated pillars: optimization of red blood cell mass through targeted hematinic therapy, minimization of blood loss, and enhancement of the patient’s physiological tolerance to anemia [5,6,7,8]. By integrating these components into perioperative practice, PBM reduces transfusion requirements and improves clinical outcomes [7,8]. The COVID-19 pandemic provided a unique opportunity to evaluate the role of PBM not only in routine clinical practice but also in maintaining healthcare system resilience during crises. Insights gained during this period highlight the importance of proactive anemia management and blood conservation strategies in supporting sustainable surgical care under conditions of limited resources. This narrative review examines contemporary alternatives to ABT within the PBM framework in surgical patients and explores the key lessons derived from the COVID-19 pandemic that may inform the future implementation of PBM programs. This narrative review focuses on the integration of PBM principles into perioperative patient care and provides a clinically oriented synthesis of anemia management in the context of the COVID-19 pandemic.

## 2. Risks and Complications Associated with Allogeneic Blood Transfusion (ABT)

Allogeneic blood transfusion is routinely used worldwide to correct severe anemia in hemodynamically unstable patients and to manage perioperative anemia, a well-recognized contributor to morbidity and mortality in surgical populations [9,10]. The primary objective of ABT is the rapid restoration of hemoglobin levels to ensure adequate tissue oxygenation. Although often lifesaving, ABT requires careful patient selection because transfusion exposure is associated with a spectrum of adverse outcomes. In contemporary clinical practice, blood transfusion typically refers to blood component administration: red blood cell concentrate, plasma, and platelets, rather than whole blood administration. This component-based approach enables a more targeted correction of specific deficiencies, acting in accordance with PBM principles. Multiple studies demonstrate a dose-dependent relationship between ABT and the inherent risk of complications, including increased infection risk, cancer recurrence, coagulopathies, and alloimmunization [9,10,11,12]. In oncologic surgical patients, ABT has been associated with elevated risks of thrombotic and embolic events, likely mediated by inflammatory and coagulation activation [9,13]. Transfusion-related immunomodulation may impair immune surveillance and create conditions favorable to tumor progression, increasing the likelihood of both cancer recurrence and postoperative bacterial infections [13,14]. For example, Gunka et al. reported that transfusion of three or more units of red blood cell concentrate during colorectal cancer surgery was associated with poorer overall survival, reduced disease-free survival, and increased distant recurrence [15]. Postoperative bacterial infection represents another major concern. A meta-analysis by Hill et al., including 13,152 patients, demonstrated a higher incidence of postoperative infections in transfused surgical patients [14]. This increased risk is primarily attributed to transfusion-related immunomodulation rather than the failure to maintain aseptic conditions during surgery. Similarly, a large retrospective cohort study of 8098 patients found that perioperative ABT was associated with increased risks of surgical site infection, pneumonia, bloodstream infection, intensive care admission, unplanned reoperation, prolonged hospitalization, and mortality [12]. A further meta-analysis of 21,770 patients undergoing total hip and knee arthroplasty confirmed ABT as a significant risk factor for surgical-site infection [16]. While transmission of viral infections such as hepatitis or HIV has markedly declined due to advances in donor selection, as well as the marked improvement in blood screening and testing protocols for different bloodborne pathogens [17,18], non-infectious transfusion complications remain clinically significant. These include transfusion-related acute lung injury (TRALI), transfusion-associated circulatory overload (TACO), transfusion-related immunomodulation (TRIM), and alloimmunization [17,19]. Among these, TRALI continues to be a leading cause of transfusion-related morbidity and mortality despite ongoing preventive measures [20]. Because many transfusion recipients are physiologically vulnerable, cardiopulmonary complications may have severe or fatal consequences. Storage-related changes in blood products constitute an additional safety concern. Prolonged storage has been linked to the accumulation of pro-inflammatory mediators and biochemical alterations that may contribute to acidosis, hemolysis, and membrane changes in red blood cells, potentially increasing complication rates [21]. The COVID-19 pandemic further emphasized the importance of maintaining rigorous transfusion safety standards while managing constrained blood supplies. Although the current risk of transmitting known bloodborne pathogens is extremely low under existing screening guidelines, emerging infectious threats remain a potential challenge due to incomplete knowledge of transmission pathways and limited early detection capacity [22].

## 3. Perioperative Patient Blood Management (PBM)

The three pillars that represent the core principles of PBM are optimization of red blood cell mass and hematocrit levels to ensure adequate tissue perfusion, minimization of blood loss, and enhancement of physiological tolerance to anemia. PBM represents a complex, multimodal approach tailored to individual patient characteristics and needs and requires coordinated multidisciplinary collaboration for effective perioperative anemia management [7]. A growing body of evidence indicates that early identification and treatment of perioperative anemia are associated with improved surgical outcomes and reduced complication rates [23]. A structured diagnostic approach is essential for accurate classification and targeted management of perioperative anemia [24]. Initial laboratory evaluation should include complete blood count, reticulocyte count, serum ferritin, transferrin saturation (TSAT), and markers of inflammation such as C-reactive protein [24]. Iron deficiency anemia is typically characterized by low ferritin (<30 μg/L) and reduced TSAT, whereas anemia of inflammation may present with normal or elevated ferritin in the presence of increased inflammatory markers [25]. In equivocal cases, additional parameters such as reticulocyte hemoglobin content and soluble transferrin receptor levels can improve diagnostic accuracy [24,25]. Assessment of vitamin B12 and folate status is recommended in macrocytic anemia, while evaluation of renal function is essential in suspected anemia of chronic kidney disease. Early identification of anemia etiology enables targeted therapy and is a cornerstone of effective patient blood management programs [24].

### 3.1. Anemia in Surgical Patients

Preoperative anemia is highly prevalent among surgical patients, with reported rates reaching up to 75% depending on population characteristics and diagnostic criteria [26]. Globally, nearly 30% of the population is affected by anemia, with a higher burden observed in women [3]. Prevalence varies according to age, comorbidities, and underlying disease. Anemia can be broadly classified according to reticulocyte count into hypoproliferative and hyperproliferative forms and further categorized by mean corpuscular volume as microcytic, normocytic, or macrocytic [27]. In patients scheduled for surgery, iron deficiency and anemia of chronic inflammation represent the most common etiologies. When these causes do not adequately explain the clinical picture, additional investigations—including assessment of vitamin B12 and folate status, lactate dehydrogenase levels, and evaluation for chronic kidney disease, hemolysis, or malabsorption—are warranted [26,27]. Less common but clinically relevant causes include inherited forms of anemia, such as hemoglobinopathies and bone marrow failure syndromes. These should be considered in selected patients. In these conditions, transfusion may remain an essential therapeutic component, particularly in severe or symptomatic cases, and requires individualized management strategies. In hemolytic anemia, management is primarily directed toward the underlying cause, including immune-mediated mechanisms, infections, or enzymatic defects. In these cases, transfusion is not routinely preferred and is reserved for severe or life-threatening anemia, as it does not address the underlying pathophysiological pathways. Iron deficiency remains the leading cause of anemia worldwide and may be absolute or functional [25]. Nutritional deficits, poverty, and broader socioeconomic determinants also play a major role in the global burden of anemia, particularly in vulnerable populations. Absolute iron deficiency results from an imbalance between iron intake and physiological requirements. Functional iron deficiency occurs when iron stores are sufficient, but iron mobilization and erythropoiesis are impaired, frequently in the context of chronic inflammatory conditions. Overexpression of hepcidin in inflammatory states inhibits iron release from macrophages and hepatocytes and reduces intestinal absorption, contributing to anemia of chronic disease [25]. Effective anemia correction requires an understanding of the underlying mechanisms regulating erythropoiesis, iron metabolism, and inflammatory signaling. Targeted therapy should therefore be guided by hematologic evaluation rather than hemoglobin concentration alone. In patients with malabsorption syndromes or functional iron deficiency, parenteral iron supplementation may be necessary [25,28,29]. Inadequate absorption or intake of vitamin B12 or folate can similarly lead to macrocytic anemia. Hemoglobin concentration remains the standard parameter for diagnosing and grading anemia. According to World Health Organization criteria, anemia is defined as hemoglobin <12 g/dL in non-pregnant adult women and <13 g/dL in adult men. In the perioperative setting, patients scheduled for major surgery with hemoglobin levels below 13 g/dL should generally be considered anemic regardless of sex [23]. A structured preoperative assessment is essential to identify and correct abnormalities in hematologic homeostasis [24]. This evaluation should include personal and family histories of bleeding disorders or thrombotic events; review of medications that may affect coagulation—including antiplatelet and anticoagulant agents, nonsteroidal anti-inflammatory drugs, selective serotonin reuptake inhibitors, and certain herbal supplements—and a focused physical examination for signs of coagulopathy. Assessment of risk factors for organ ischemia is also important in guiding transfusion decisions. Serum ferritin is a key diagnostic marker of iron deficiency anemia, with values below 30 μg/L demonstrating high sensitivity and specificity [25]. When iron deficiency is excluded, further evaluation of renal function, including serum creatinine measurement, is recommended. Effective perioperative anemia management strategies aimed at reducing transfusion requirements include oral or intravenous iron supplementation, correction of nutritional deficiencies, and administration of erythropoiesis-stimulating agents. Iron therapy is particularly appropriate for elective procedures in which sufficient time is available for hematologic optimization [24,30]. In acute settings, such as traumatic blood loss or emergency surgery, immediate correction of anemia and hemorrhage control are of paramount importance and should be addressed immediately. In these situations, transfusion may be unavoidable, and PBM principles focus on minimizing unnecessary exposure while ensuring hemodynamic stability and adequate tissue oxygenation [31].

**Figure 1:**
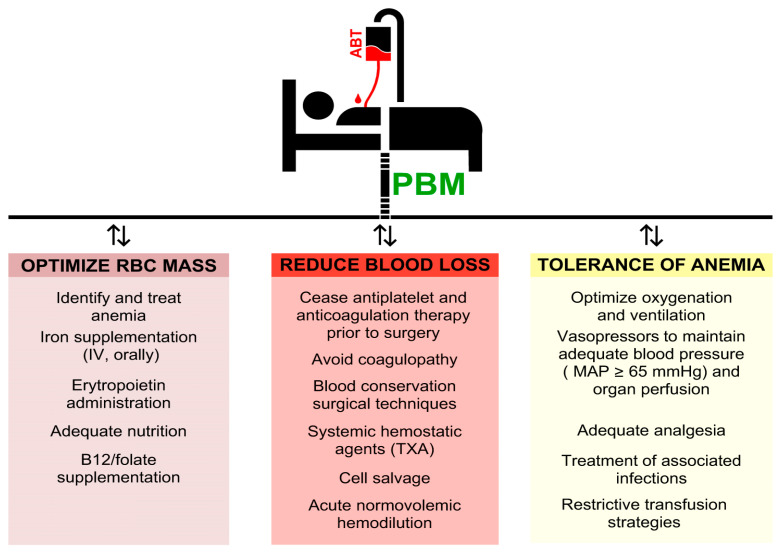
Overview of the patient blood management approach. Arrows indicate the relationship between the three pillars of patient blood management and their role in perioperative anemia management.

A structured diagnostic framework complements the PBM model illustrated in Figure 1 and supports targeted identification of perioperative anemia etiologies.

### 3.2. Anemia Correction

Correction of perioperative anemia represents a central objective of PBM. The COVID-19 pandemic underscored the vulnerability of blood supply systems and reinforced the importance of proactive anemia management as an alternative to transfusion-dependent strategies [1,32]. Because iron deficiency anemia (IDA) is the most frequent cause of perioperative anemia, iron supplementation constitutes a cornerstone of PBM.

#### 3.2.1. Iron Supplementation

Iron may be administered orally or intravenously, with intravenous (IV) formulations providing faster and more predictable hematologic responses [24,33,34]. Oral iron remains an option for stable patients undergoing elective procedures, whereas IV iron is preferred when rapid correction is required. Decisions regarding transfusion versus iron therapy should consider anemia severity, symptom burden, timing of surgery, therapeutic availability, and patient preferences [24,35]. Screening for iron deficiency ideally occurs at least four weeks before surgery to allow sufficient time for optimization [30]. Although oral supplementation is widely used in outpatient settings, its effectiveness is limited by gastrointestinal adverse effects, variable absorption, and the prolonged duration required to restore iron stores [36]. Up to 60% of patients report gastrointestinal intolerance, which may compromise adherence [37]. Consequently, oral iron is generally reserved for mild anemia, while IV iron is favored in moderate to severe cases or when rapid correction is necessary [33,35]. Growing evidence supports the perioperative benefits of IV iron therapy. Observational and interventional studies demonstrate reduced transfusion requirements, increased preoperative hemoglobin levels, and shorter hospital stays when supplementation is initiated at least 7–10 days before surgery [29,38,39,40]. Oral iron failure should prompt evaluation for malabsorption syndromes, ongoing blood loss, or poor adherence, with transition to parenteral therapy when appropriate [28]. Early identification of iron deficiency—ideally 2–3 weeks preoperatively—remains critical for effective optimization [7,24].

#### 3.2.2. Vitamin B12 and Folate Supplementation

Deficiencies of vitamin B12 and folate result in macrocytic anemia and should be corrected when identified. Treatment duration and administration route depend on the underlying etiology, and lifelong supplementation may be required in selected patients. Hematologic improvement can begin within approximately 10 days of therapy initiation [23,41].

#### 3.2.3. Erythropoiesis-Stimulating Agents (ESAs)

Erythropoiesis-stimulating agents are indicated in selected patients with anemia unresponsive to iron therapy or in anemia unrelated to nutritional deficiency, such as chronic kidney disease. Concomitant iron supplementation is essential to support effective erythropoiesis [23]. Current guidelines recommend ESAs, iron therapy, or combined treatment for anemia associated with chronic kidney disease [42]. Evidence suggests that perioperative ESA use—particularly when combined with IV iron, vitamin B12, and folate—can reduce postoperative transfusion requirements and increase preoperative hemoglobin levels [43,44]. This strategy may also benefit selected non-anemic patients undergoing procedures with anticipated major blood loss. However, ESA therapy is associated with cardiovascular and thromboembolic risks, particularly in patients diagnosed with various malignancies [45]. Careful patient selection and monitoring are mandatory. ESAs should be avoided in individuals with recent severe thromboembolic events or significant cerebrovascular disease, and potential interactions with antihypertensive agents should be considered [46]. Overall, ESA administration requires individualized risk–benefit assessment. From a hematologic perspective, careful patient selection and monitoring are essential to balance erythropoietic stimulation with thrombotic risk, particularly in populations with underlying inflammatory or neoplastic conditions.

### 3.3. Blood Loss Minimization

#### 3.3.1. Balancing Thrombotic and Bleeding Risk

Perioperative management of antiplatelet and anticoagulant therapy requires careful evaluation of competing thrombotic and bleeding risks [47,48,49]. Risk assessment incorporates patient-specific factors—including prior bleeding, malignancy, thrombophilia, and thrombocytopenia—as well as procedural characteristics. Clinical scoring systems such as BleedMAP can support decision-making [50]. Laboratory evaluation may include prothrombin time, activated partial thromboplastin time, fibrinogen levels, and viscoelastic assays. International normalized ratio monitoring allows adjustment of vitamin K antagonist therapy, and low-dose oral vitamin K can correct supratherapeutic values to prevent surgical delays [47]. Viscoelastic hemostatic assays such as thromboelastography and rotational thromboelastometry provide rapid global assessment of coagulation and are increasingly used in severe bleeding [48]. Patients with inherited bleeding disorders require specialized perioperative planning and correction of hemostatic defects whenever possible [51,52]. Acute trauma-associated coagulopathy represents a distinct entity requiring rapid intervention. Early administration of antifibrinolytics, particularly tranexamic acid (TXA), reduces mortality in bleeding trauma patients and significantly decreases transfusion requirements in orthopedic and cardiac surgery without increasing thromboembolic complications [48,53,54]. COVID-19-associated hypercoagulability introduced additional complexity to perioperative management. Patients with perioperative SARS-CoV-2 infection exhibit increased thrombotic complications and mortality, necessitating heightened vigilance [55,56].

#### 3.3.2. Surgical Techniques to Reduce Bleeding

Surgical technique and effective local hemostasis remain fundamental to blood conservation. Appropriate patient positioning can influence venous pressures and intraoperative bleeding [48]. Minimally invasive approaches, including laparoscopic and robotic surgery, are consistently associated with reduced blood loss and faster recovery compared with open procedures [8]. In high-risk settings such as cardiac surgery, validated bleeding risk scores can guide preventive strategies and resource allocation [48,57]. Standardized intraoperative hemostatic protocols, including meticulous surgical dissection, judicious use of electrocautery, and application of topical hemostatic agents, further contribute to reducing blood loss. The adoption of enhanced recovery after surgery (ERAS) pathways also supports hemodynamic stability and optimized coagulation status. Close communication between surgical and anesthesia teams is essential to maintain normothermia, prevent acidosis, and avoid coagulopathy, all of which directly influence bleeding risk.

#### 3.3.3. Cell Salvage

Intraoperative cell salvage is recommended for procedures with anticipated major blood loss [58]. Reinfused salvaged blood lacks clotting factors and platelets, and adjunctive hemostatic therapy may be required. Meta-analyses demonstrate that cell salvage reduces allogeneic transfusion exposure without increasing complication rates and represents a cost-effective alternative in appropriate settings [59,60]. Although theoretical concerns exist regarding tumor cell dissemination, current evidence does not demonstrate increased oncologic risk in carefully selected patients. Modern cell salvage systems incorporate filtration and washing processes that reduce contamination with debris, activated cytokines, and free hemoglobin. In oncologic surgery, the use of leukocyte depletion filters further mitigates theoretical concerns regarding tumor cell reinfusion. Appropriate patient selection and institutional protocols are important to maximize safety and cost-effectiveness.

#### 3.3.4. Acute Normovolemic Hemodilution

Acute normovolemic hemodilution involves the removal of autologous blood immediately before surgery with simultaneous volume replacement. When integrated into multimodal PBM strategies, it can reduce transfusion requirements without increasing perioperative complications [61,62]. Benefits have been reported in cardiac surgery, including improved perfusion during cardiopulmonary bypass due to reduced blood viscosity [63]. The theoretical advantage of acute normovolemic hemodilution lies in reducing red blood cell loss during surgical bleeding while preserving autologous blood for reinfusion. Maintenance of normovolemia ensures adequate tissue perfusion and oxygen delivery despite temporary hemodilution. Careful patient selection is essential, particularly in individuals with limited cardiopulmonary reserve.

### 3.4. Physiological Tolerance to Anemia

Compensatory physiological mechanisms allow temporary adaptation to anemia by optimizing oxygen delivery. Supportive strategies—including supplemental oxygen, maintenance of adequate perfusion (with a target mean arterial pressure ≥ 65 mmHg), using vasopressors, when necessary, effective analgesia, and infection prevention—can enhance tolerance to reduced hemoglobin levels and decrease tissue oxygen demand [31,64]. From a physiological perspective, compensatory mechanisms include increased cardiac output, redistribution of blood flow toward vital organs, and enhanced oxygen extraction at the tissue level. Optimization of these adaptive responses requires maintenance of normovolemia, normothermia, and adequate hemoglobin–oxygen affinity. Avoidance of hemodilution beyond tolerable limits, correction of acidosis, and prevention of hypoxia are critical components of supportive management. In selected patients, individualized transfusion thresholds should be guided, not only by hemoglobin concentration, but also by clinical signs of impaired oxygen delivery, including lactate levels, mixed venous oxygen saturation, and hemodynamic instability.

## 4. Lessons Learned from the COVID-19 Pandemic for Patient Blood Management

The COVID-19 pandemic exposed critical vulnerabilities in global healthcare systems and provided important lessons about maintaining safe and sustainable patient care. The pandemic also exposed a persistent tendency toward ‘liberal’ transfusion practices in some high-income healthcare systems, where transfusion has historically been used as a rapid corrective measure for anemia. This “replacement-based” approach contrasts with contemporary PBM principles, which emphasize restrictive, physiology-guided transfusion strategies and targeted correction of underlying causes of anemia. Several key lessons emerged that extend beyond the immediate crisis and have long-term implications for perioperative practice. First, the fragility of blood supply systems became evident. Lockdowns, social distancing measures, and reduced donor availability caused significant disruptions in blood donation services worldwide, resulting in shortages of blood products. This experience demonstrated that healthcare systems heavily dependent on allogeneic transfusion are particularly vulnerable during large-scale crises. Institutions with established PBM programs were better positioned to preserve limited resources by reducing transfusion demand through proactive anemia management and blood conservation strategies. Second, early identification and correction of perioperative anemia proved essential. The pandemic reinforced the importance of systematic preoperative screening pathways and timely treatment using iron therapy, nutritional supplementation, and selected use of erythropoiesis-stimulating agents. Hospitals that implemented structured PBM protocols were able to maintain surgical activity while minimizing transfusion exposure and associated complications. Third, multidisciplinary collaboration emerged as a cornerstone of effective PBM implementation. The crisis accelerated cooperation between anesthesiologists, surgeons, hematologists, and transfusion specialists. This integrated model improved clinical decision-making, optimized resource allocation, and demonstrated that PBM functions most effectively when embedded within institutional culture rather than applied as an isolated intervention. Fourth, PBM contributes to healthcare system resilience and crisis preparedness. Beyond improving individual patient outcomes, PBM reduces reliance on scarce blood resources, shortens hospitalization, and lowers complication rates. These characteristics align PBM with broader public health strategies aimed at strengthening healthcare infrastructure and preparedness for future emergencies. Finally, the pandemic highlighted the need for standardized PBM implementation and clinician education. Variability in PBM adoption across institutions revealed disparities in preparedness. Establishing uniform guidelines, investing in training, and integrating PBM into routine perioperative care are essential steps toward sustainable improvement. Together, these lessons position PBM not only as a clinical strategy for optimizing perioperative outcomes but also as a framework for enhancing healthcare system adaptability in the face of future global challenges. These considerations are particularly relevant for low- and middle-income countries, where blood supply systems are often affected by structural challenges including limited governance, insufficient strategies for donor motivation, fragmentation of transfusion services, and inconsistent access to safe blood products. In these settings, the pandemic further amplified existing shortages and widened disparities between resource-rich and resource-limited healthcare systems. Thus, patient blood management represents an evidence-based, multidisciplinary approach that optimizes the utilization of healthcare resources, while simultaneously serving as a critical strategy for mitigating disparities between high- and low-resource health systems, where limited availability and inequitable access to blood products necessitate more efficient, patient-centered alternatives.

## 5. Future Perspectives in Patient Blood Management

**Figure 2:**
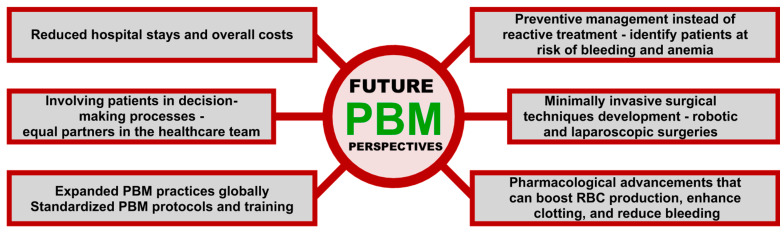
Conceptual overview of future directions in patient blood management.

The progressive adoption of patient blood management as a standard component of perioperative care is expanding across diverse clinical settings. The COVID-19 pandemic emphasized the necessity of strengthening blood supply resilience and accelerating innovation in anemia management and blood conservation strategies. Future developments in PBM are likely to be driven by advances in technology, personalized medicine, and data integration. Digital health tools and artificial intelligence (AI) systems have the potential to transform PBM by enabling predictive risk modeling, automated screening for perioperative anemia, and individualized transfusion decision support. Integration of large clinical datasets into AI-assisted platforms may improve early identification of high-risk patients and optimize resource allocation. Advances in pharmacologic therapies and iron formulations are expected to refine perioperative anemia correction. Ongoing research into novel erythropoiesis-modulating agents and targeted anti-inflammatory therapies may further expand treatment options. In parallel, continued evolution of minimally invasive surgical techniques and enhanced recovery protocols is likely to reduce intraoperative blood loss and transfusion requirements. Standardization of screening algorithms and implementation of evidence-based PBM protocols remain critical priorities. Education and training programs for healthcare professionals are crucial for the successful implementation of patient blood management programs. Structured education programs are essential for the long-term sustainability and consistent application of PBM strategies across diverse healthcare settings. Improving awareness of evidence-based transfusion practices, increasing adherence to clinical guidelines, and fostering multidisciplinary collaboration are key components in translating PBM principles into routine clinical practice. Development of validated risk stratification tools and institutional PBM pathways can facilitate early intervention and shift clinical focus from reactive transfusion to proactive anemia prevention. Such strategies may contribute to shorter hospital stays, lower healthcare costs, and improved patient outcomes. Collectively, these perspectives suggest that the future of PBM lies in the integration of technological innovation, multidisciplinary collaboration, and preventive perioperative care models aimed at sustainable optimization of blood resources. These future directions are summarized in Figure 2.

## 6. Conclusions

Rather than introducing PBM as a novel concept, this review provides a contemporary synthesis that integrates established PBM principles with recent evidence and insights derived from the COVID-19 pandemic, with particular relevance for perioperative hematology practice and resource-limited settings. The COVID-19 pandemic exposed the structural vulnerability of global blood supply systems and accelerated recognition of patient blood management (PBM) as a strategic, evidence-based alternative to transfusion-centered perioperative care [1,65]. Accumulating clinical evidence demonstrates that PBM improves surgical outcomes by reducing morbidity, mortality, and transfusion exposure while simultaneously lowering healthcare costs and preserving limited blood resources. More importantly, PBM reframes perioperative anemia from an acute transfusion trigger to a modifiable clinical condition requiring proactive, mechanism-driven management. This paradigm shift places hematologic assessment at the center of perioperative decision-making, emphasizing mechanism-based diagnosis and targeted correction of anemia. From a hematologic perspective, perioperative anemia should be systematically screened, accurately characterized, and treated through targeted interventions integrated into routine surgical pathways. Early identification of iron deficiency and other correctable etiologies, timely administration of intravenous iron when rapid optimization is required, and selective use of erythropoiesis-stimulating strategies represent essential components of contemporary perioperative care. Effective implementation of these measures depends on close interdisciplinary collaboration and on embedding hematologic expertise within institutional PBM frameworks. Despite strong international endorsement, including recommendations from the World Health Organization, PBM adoption remains inconsistent across healthcare systems [5]. Persistent barriers—ranging from variability in guideline implementation to infrastructural constraints and entrenched transfusion practices—highlight the need for coordinated policy initiatives, clinician education, and institutional commitment to standardized PBM pathways [66,67]. Emerging paradigms increasingly advocate for individualized transfusion decisions guided not only by hemoglobin thresholds but also by physiological indicators of tissue oxygenation and perfusion [64]. Integration of digital health tools and data-driven decision support systems may further refine risk stratification and resource allocation. Ultimately, PBM represents more than a set of clinical interventions; it constitutes a systemic framework for improving patient safety and strengthening healthcare resilience. Lessons from the COVID-19 era emphasize that sustainable perioperative care requires a decisive shift toward proactive anemia management and responsible use of blood resources. For hematologists, perioperative anemia offers a critical opportunity to influence outcomes through early intervention and leadership in PBM implementation. The continued evolution of PBM will depend on its integration into routine clinical practice and on sustained commitment to evidence-based, patient-centered transfusion strategies capable of meeting future healthcare challenges.

## Data Availability

No new data were created or analyzed in this study.

## Abbreviations

The following abbreviations are used in this manuscript:

PBM	Patient blood management
ABT	Allogeneic blood transfusion
TSAT	Transferrin saturation
IDA	Iron deficiency anemia
IV	Intravenous
ESAs	Erythropoiesis-stimulating agents
TXA	Tranexamic acid
TRALI	Transfusion-related acute lung injury
TACO	Transfusion-associated circulatory overload
TRIM	Transfusion-related immunomodulation
WHO	World Health Organization
KDIGO	Kidney Disease: Improving Global Outcomes
